# Metabolites in aging and autophagy

**DOI:** 10.15698/mic2014.04.142

**Published:** 2014-04-07

**Authors:** Sabrina Schroeder, Andreas Zimmermann, Didac Carmona-Gutierrez, Tobias Eisenberg, Christoph Ruckenstuhl, Aleksandra Andryushkova, Tobias Pendl, Alexandra Harger, Frank Madeo

**Affiliations:** 1 Institute of Molecular Biosciences, University of Graz, Humboldtstrasse 50, 8010 Graz, Austria.; 2 Division of Endocrinology and Metabolism, Dept. of Internal Medicine, Medical University of Graz, Auenbruggerplatz 15, 8036 Graz, Austria.

**Keywords:** autophagy, aging, metabolism, acetyl-CoA, polyamines, amino acids

## INTRODUCTION

Autophagy, the main lysosomal degradative machinery, plays a major role in maintaining cellular homeostasis and thus a healthy state in an organism. This process recycles unnecessary or damaged material, therefore, not only providing nutrients to maintain vital cellular functions in times of starvation but also eliminating potentially harmful cellular material 1. Importantly, the autophagic rate declines with increasing age 2,3, suggesting a functional correlation between aging and autophagy. Indeed, the deregulation of autophagy is involved in the onset of various age-related diseases such as cancer, cardiomyopathy, type II diabetes, and neurodegeneration 4. Until recently, aging was regarded as an unregulated and inescapable consequence of the accumulation of incidental damage in macromolecules and/or organelles. However, the discovery of multiple ways to extend the lifespan in a variety of different model organisms, e.g., by genetic and pharmacological means, developed the formulation of alternative aging theories that consider aging as a molecular program 5. Indeed, the last years have provided important insights into the networks that control aging and have thus highlighted the interconnected nature of aging and various cellular processes. For instance, the process of aging is intimately coupled to metabolic processes 6, in particular to energy metabolism and nutrient availability. Nevertheless, specific metabolites that affect aging and autophagy remain poorly described.

## NUTRIENT AVAILABILITY CONTROLS AUTOPHAGY AND AGING VIA ENERGY METABOLITES 

As sensors of the current environmental status, nutrient signaling pathways represent central aging regulators. For instance, individual interventions in the insulin/insulin-like growth factor 1 (IGF-1), Ras, protein kinase A (PKA), target of rapamycin (Tor), or protein kinase B (SCH9/Akt) pathways have been shown to extend lifespan in various organisms, including mammals 7,8,9. Caloric restriction (CR) requires autophagy for lifespan extension 10,11,12 and CR-mediated autophagy induction follows molecular pathways that are shared with those known to affect aging, such as Tor, SCH9/Akt, or IGF-1 13. Furthermore, the AMP-activated protein kinase (AMPK) serves as a metabolic radar sensing changes in the AMP/ATP ratio and is conserved in the majority of eukaryotic species, and has also been established as a checkpoint for growth control and autophagy regulation 14. Consistently, several studies have revealed a connection between the AMP/ATP ratio, autophagic flux rates, senescence, and disease 15,16. Noteworthy, early studies on rat hepatocytes also suggested that the execution of autophagy depends on energy availability since inhibition of ATP production stalls autophagic flux 17. Other pivotal energy sources like butyrate, an essential energy component in the colon, and second messengers such as cAMP, which might also be implicated in Ras/PKA-mediated lifespan modulation in various organisms, were identified as potential autophagy mediators 18,19. This argues for a decisive function of nutrient signaling and energy metabolites during aging and its associated processes.

**Figure 1:**
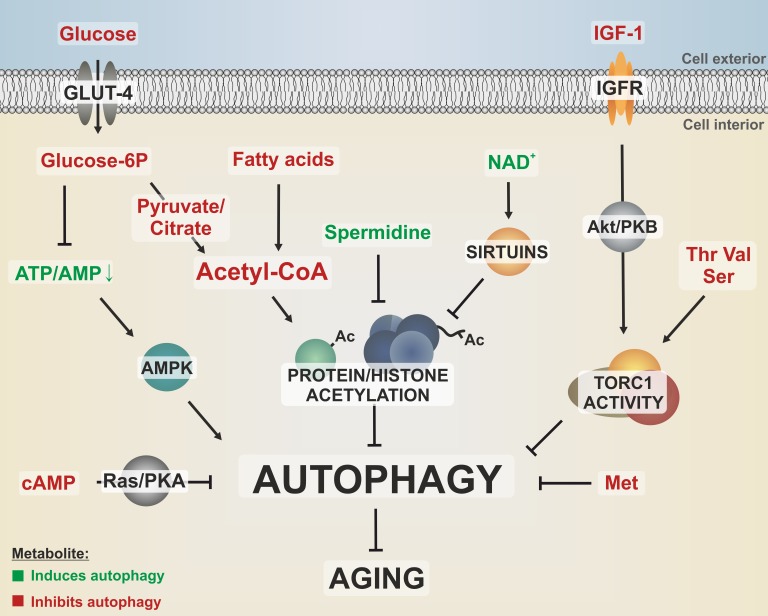
FIGURE 1: Different metabolites converge on pathways that regulate autophagy and aging. Dietary nutrients like glucose, amino acids and fatty acids as well as growth signaling by IGF-1 activate nutrient-sensing kinases, like the target of rapamycin complex 1 (TORC1), which stalls autophagy via phosphorylation of downstream targets. Furthermore, autophagy is negatively regulated by the Ras/PKA pathway, which responds to nutrient availability by sensing intracellular cAMP levels. The cellular energy status is reflected by the ATP/AMP ratio, which is sensed by the autophagy activator AMPK. Methionine downregulates autophagy during aging in a yet to be elucidated fashion. The central energy intermediate acetyl-CoA integrates metabolites from glycolysis, β-oxidation or respiration and fuels acetylation of proteins such as histones, resulting in decreased autophagic flux. All these autophagy-limiting metabolic pathways have been linked to an accelerated aging phenotype. In contrast, polyamines, like spermidine, reduce protein acetylation, thereby promoting autophagy and longevity. Potential crosstalks between protein acetylation and nutrient sensing kinase signaling are yet to be elucidated. GLUT-4, glucose transporter 4; IGFR, IGF-1 receptor; Ac, acetyl-group; amino acids are indicated by three-letter code.

## THE ENERGY METABOLITE ACETYL-CoA SUPPRESSES AUTOPHAGY VIA PROTEIN ACETYLATION

In the yeast *S cerevisiae*, glucose is the preferred carbon source for fueling energy metabolism and its fermentation produces acetate and ethanol that is used for subsequent respiration after the diauxic shift. Both intermediates are released into the medium and have been attributed a role in limiting yeast chronological life span 20, but considered a pro-aging factor specific for yeast 21. However, acetate is an important metabolite that is involved in central processes such as acetyl-Coenzyme A (acetyl-CoA) production. Thus, its metabolic fate rather than a simply extracellular toxicity may (at least in part) be responsible for its impact on lifespan. Consequently, acetate metabolism may also contribute to aging in higher eukaryotes, possibly via its impact on protein acetylation through acetyl-CoA generation and subsequent control of cellular function. Energy metabolites that can derive in the production of acetyl-CoA, such as citrate, pyruvate and fatty acids, were shown to be deregulated in senescence-accelerated mice 22. We could recently show that (nucleo-)cytosolic acetyl-CoA, in fact, serves as a modulator of longevity, suppressing starvation and age-associated autophagy in a variety of phyla 23,24. This function might be due to the fact that acetyl-CoA is the only donor for acetylation reactions and both, protein acetylation and epigenetic chromatin modifications, have repeatedly been linked to the regulation of aging and autophagy 23,25,26. For instance, the highly conserved protein family of NAD+-dependent histone deacetylases and ADP ribosylases (sirtuins) has been connected to aging modulation 27,28 and lifespan extension upon CR 29. Activation of histone deacetylases, such as sirtuins, has therefore been extensively studied for its capability to combat aging or age-associated pathologies. Interestingly, the lifespan-extending effects of sirtuin activation by CR or pharmacological interventions depend on the induction of autophagy 11. The dependency on NAD+ as a cofactor and the tight connections between sirtuins, longevity and autophagy induction have led to the hypothesis that sirtuins act as metabolic sensors that promote mitochondrial maintenance 30. Notably, nicotinamide metabolism has become an intensively investigated target for drug discovery against a variety of human diseases, including age-associated pathologies such as cancer or neurodegeneration 31.

## POLYAMINE METABOLISM LEAVES A REGULATORY FINGERPRINT AT HISTONE MODIFICATION SITES

The levels of polyamines, a class of ubiquitously occurring small basic polycations, decline with progressing age in various organisms, including humans 32, yeast 25 and plants 33. Intriguingly, external application of a specific polyamine (spermidine) counteracts cell death during aging and improves the lifespan of yeast, flies, worms, and human immune cells in an autophagy-dependent manner 25. Furthermore, it causes a reduction of oxidative stress in mice. In addition, more recent studies also suggest longevity-promoting effects in mammals 34. Importantly, spermidine treatment appears to be associated with both histone hypoacetylation caused by inhibition of histone acetyl transferases 25 and deacetylation of cytosolic proteins 35. However, it has also been hypothesized that polyamines influence histone acetylation in dependence of the histones’ own acetylation status 36 and massive polyamine catabolism has even been shown to deplete the acetylation co-factor acetyl-CoA 37. Possibly, the involvement of spermidine in the biosynthesis of the methyl-group donor S-adenosyl-methionine (SAM) 38 could also culminate in regulatory methylation reactions, such as chromatin silencing. Thus, polyamines may influence chromatin structure and protein acetylation via multiple mechanisms. Given the aforementioned contribution of acetyl-CoA to aging and autophagy modulation, it is tempting to speculate that spermidine functions include a down-titration of the intracellular acetyl-CoA pool and thus a rearrangement of the metabolic state.

## SPECIFIC AMINO ACID STARVATION INDUCES AUTOPHAGY AND PROLONGS LIFESPAN 

In line with the vast importance of nutrient signaling during aging, amino acid metabolism has an important impact on eukaryotic aging and its related diseases. The levels of specific amino acids like tryptophane, methionine, arginine, or leucine has often been suggested to (positively or negatively) influence the autophagy pathway and impact aging in different eukaryotes 39,40,41,42. Indeed, we could recently demonstrate that limitation of the amino acid methionine enhances yeast chronological lifespan. Intriguingly, this lifespan extension requires autophagy-dependent vacuolar acidification 43. In the same line, vacuolar acidification also elongates replicative lifespan of yeast, where it protects mitochondria most probably via an improvement of the vacuolar amino acid storage function 44. In yeast, serine, threonine and valine promote cellular senescence probably via activation of the Sch9/TOR pathway and subsequent inhibition of the protein kinase Rim15p, which guides anti-aging stress response pathways 45. Altogether, these examples suggest that the intake or limitation of specific amino acids is a determining factor during aging, though the mechanistic specificities are expected to be complexly regulated, especially at the organismal level.

## CONCLUSION 

A variety of potential metabolic controllers of autophagy and health span have already been proposed. However, precise strategies to target the correlating pathways (e.g., by nutrition patterns) remain to be elucidated in more detail. For example, it would be of great interest to determine if special diets that include the limitation of (defined) amino acids or the uptake of certain polyamines, like spermidine, influence the metabolism towards improved cellular conditions during aging. It also remains elusive how certain diets may affect the microbiome and in turn impact the levels of certain metabolites that have been shown to regulate cellular fitness, such as citrate, pyruvate, butyrate, or acetate. The investigation of metabolites as powerful rheostats in aging and autophagy is supported by the improvement of technologies that have opened up new possibilities to detect and trace even small molecules *in vitro* and *in vivo*. This might bring up metabolomics as a future trend for aging analyses 46. Hopefully, the findings on the impact of metabolism on aging will culminate in amended dietary guidelines that would make eating the tastiest of all medicines.
